# Supplementary figures and images for: Fermentation by Multiple Bacterial Strains Improves the Production of Bioactive Compounds and Antioxidant Activity of Goji Juice
Source: Molecules. 2019 Sep 28;24(19):3519. doi: 10.3390/molecules24193519 (PMC6804111; doi:10.3390/molecules24193519)

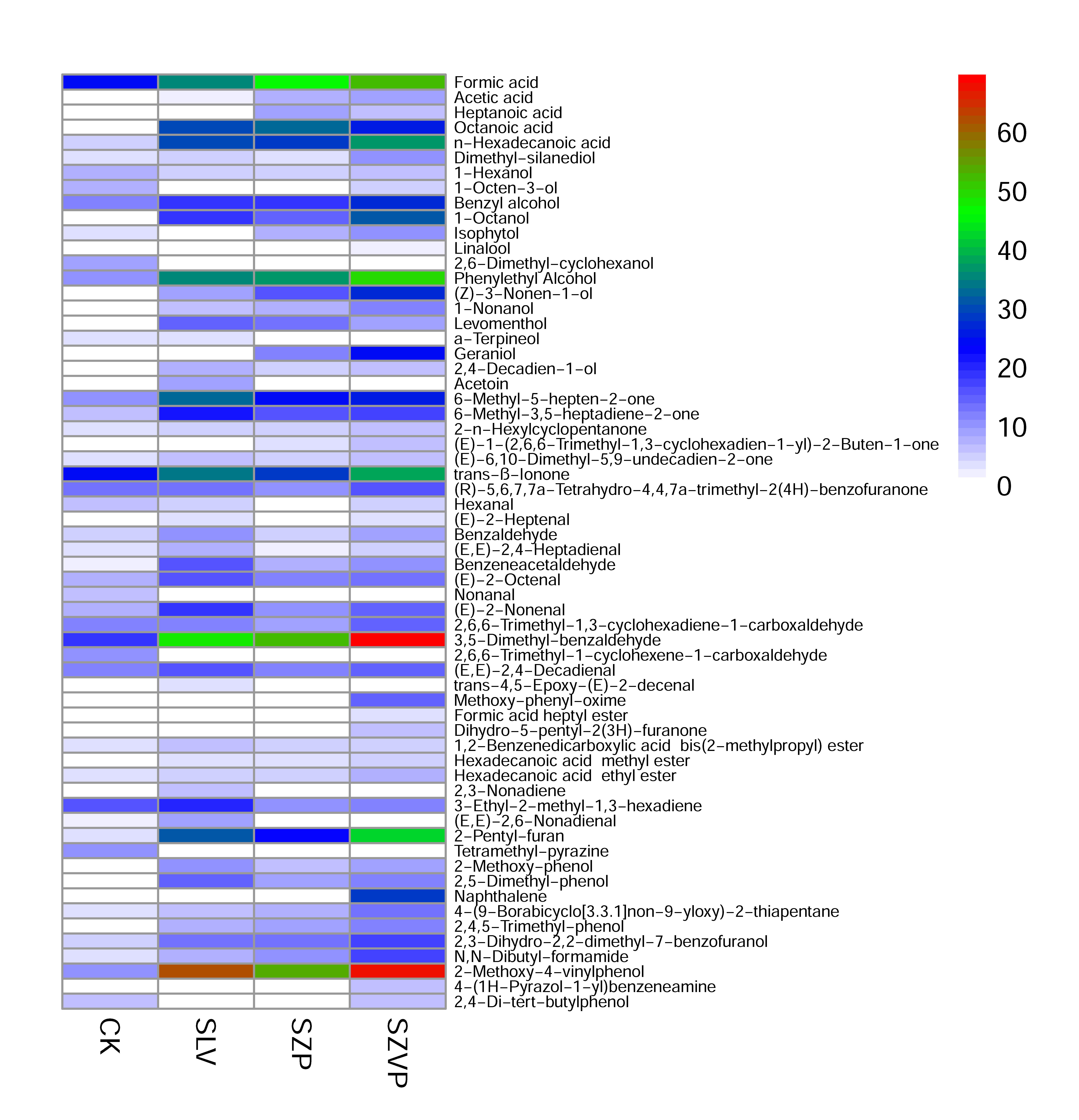

Supplement: Supplementary file 1 [file molecules-24-03519-s001.zip › Supplementary files/Figure 1..jpg]

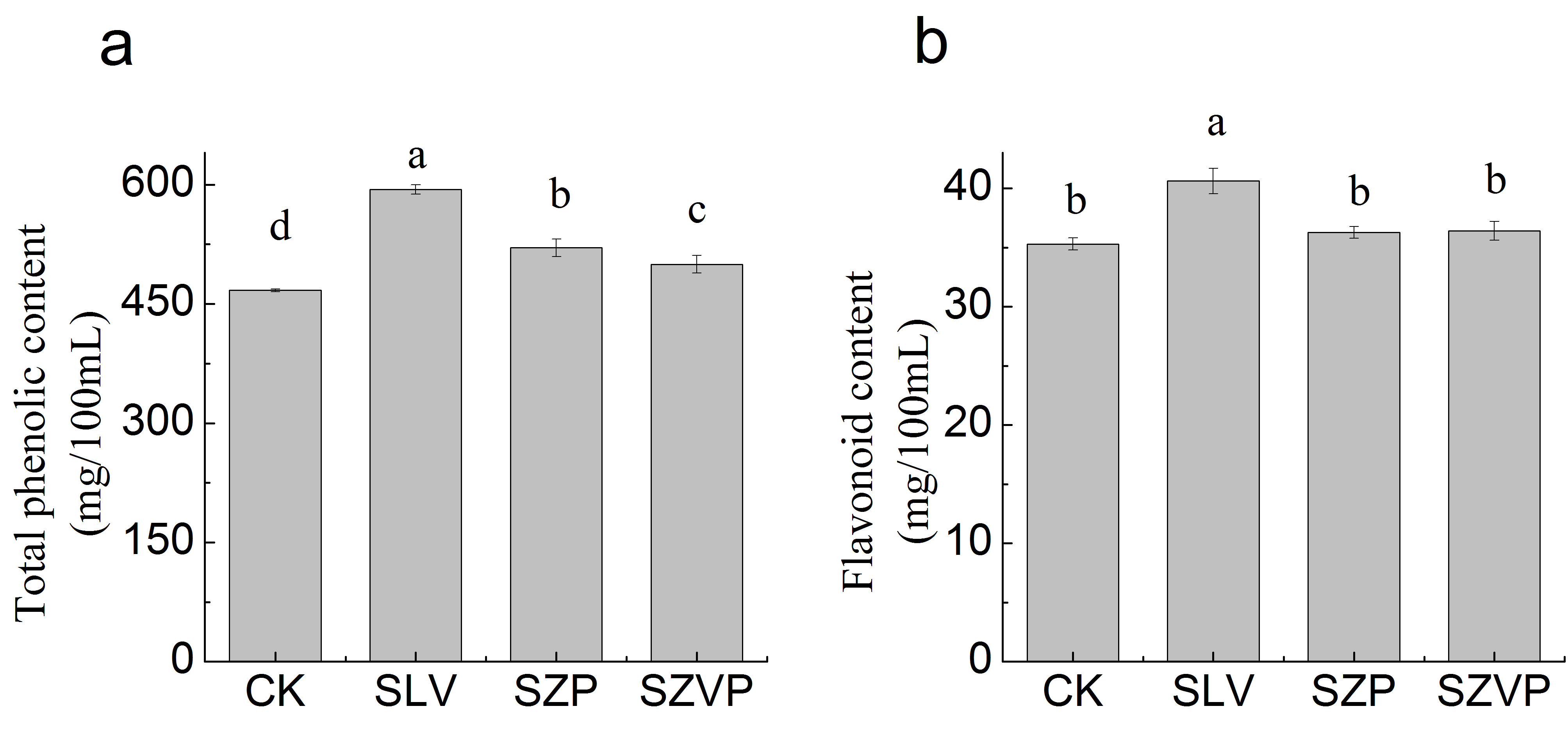

Supplement: Supplementary file 1 [file molecules-24-03519-s001.zip › Supplementary files/Figure 2..jpg]

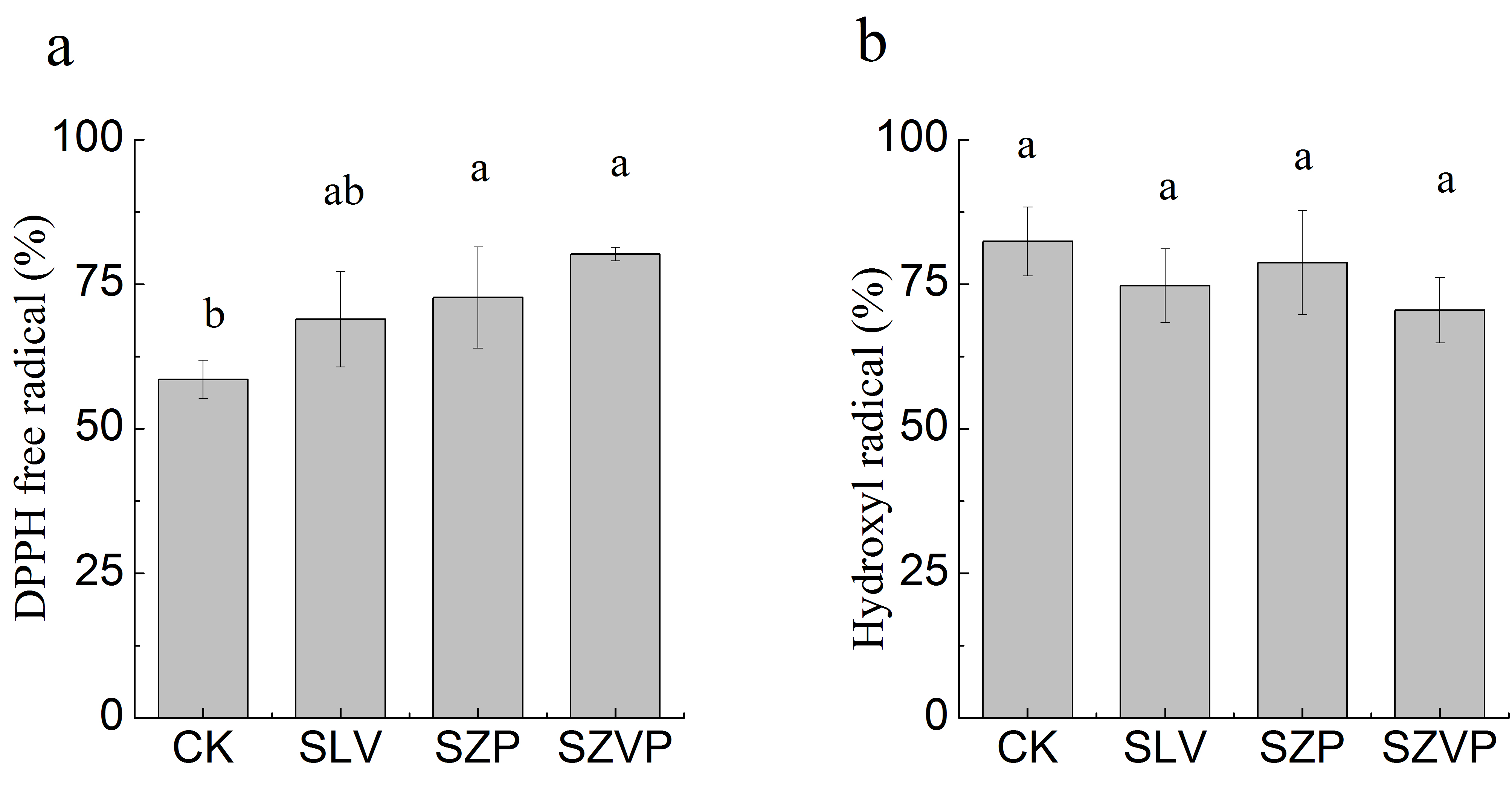

Supplement: Supplementary file 1 [file molecules-24-03519-s001.zip › Supplementary files/Figure 3..jpg]
