# Supplementary material for: Fermentation by Multiple Bacterial Strains Improves the Production of Bioactive Compounds and Antioxidant Activity of Goji Juice
Source: Molecules. 2019 Sep 28;24(19):3519. doi: 10.3390/molecules24193519 (PMC6804111; doi:10.3390/molecules24193519)
Supplement: Supplementary file 1 [file molecules-24-03519-s001.zip › Supplementary files/Supplementary table 1..doc]

Supplementary table 1. The contents of volatile compounds in the fermented goji juice by different bacteria (µg/100 g).

| **Compound** | **Time** | **RI** | **Chemical**  **formula** | **Samples** | | | |
| --- | --- | --- | --- | --- | --- | --- | --- |
|  |  |  |  | **CK** | **SLV** | **SZP** | **SZVP** |
| **Acids** |  | | | | | | |
| Formic acid | 1.894 | <800 | CH_2_O_2_ | 24.30 ± 2.00^c^ | 35.37 ± 1.98^b^ | 46.80 ± 4.28^a^ | 52.18 ± 04.56^a^ |
| Acetic acid | 2.442 | <800 | C_2_H_4_O_2_ | —^d^ | 2.54 ± 0.00^c^ | 8.00 ± 0.00^b^ | 8.89 ± 0.82^a^ |
| Heptanoic acid | 12.434 | 1077 | C_7_H_14_O_2_ | —^c^ | —^c^ | 8.40 ± 0.00^a^ | 6.06 ± 0.00^b^ |
| Octanoic acid | 15.697 | 1189 | C_8_H_16_O_2_ | —^c^ | 29.48 ± 5.03^ab^ | 32.15 ± 2.22^a^ | 25.18 ± 4.61^b^ |
| n-Hexadecanoic acid | 33.267 | 1964 | C_16_H_32_O_2_ | 4.89 ± 0.91^c^ | 29.74 ± 4.06^b^ | 28.36 ± 4.30^b^ | 36.55 ± 1.07^a^ |
| **Alcohols** |  | | | | | | |
| Dimethyl-silanediol | 3.113 | <800 | C_2_H_8_O_2_Si | 4.11 ± 0.57^b^ | 5.50 ± 1.70^b^ | 3.61 ± 0.03^b^ | 10.98 ± 1.62^a^ |
| 1-Hexanol | 6.409 | 855 | C_6_H_14_O | 8.02 ± 0.83^a^ | 5.11 ± 1.10^bc^ | 4.30 ± 0.38^c^ | 6.14 ± 1.23^b^ |
| 1-Octen-3-ol | 9.55 | 978 | C_8_H_16_O | 8.25 ± 1.49^a^ | —^c^ | —^c^ | 5.21 ± 0.00^b^ |
| Benzyl alcohol | 11.179 | 1035 | C_7_H_8_O | 11.99 ± 1.35^c^ | 18.45 ± 2.67^b^ | 18.83 ± 1.19^b^ | 27.46 ± 5.85^a^ |
| 1-Octanol | 12.25 | 1071 | C_8_H_18_O | —^d^ | 19.23 ± 1.83^b^ | 14.11 ± 1.21^c^ | 31.72 ± 1.31^a^ |
| Isophytol | 12.975 | 1096 | C_20_H_40_O | 2.89 ± 0.17^b^ | —^c^ | 8.25 ± 1.29^a^ | 10.12 ± 2.48^a^ |
| Linalool | 13.149 | 1102 | C_10_H_18_O | —^b^ | —^b^ | —^b^ | 2.23 ± 0.00^a^ |
| 2,6-Dimethyl-cyclohexanol | 13.482 | 1113 | C_8_H_16_O | 9.06 ± 0.74^a^ | —^b^ | —^b^ | —^b^ |
| Phenylethyl alcohol | 13.524 | 1114 | C_8_H_10_O | 10.31 ± 1.23^c^ | 36.26 ± 5.73^b^ | 37.04 ± 1.85^b^ | 49.4 ± 5.66^a^ |
| (Z)-3-Nonen-1-ol | 14.722 | 1155 | C_9_H_18_O | —^d^ | 9.51 ± 1.00^c^ | 15.99 ± 1.95^b^ | 27.51 ± 0.33^a^ |
| 1-Nonanol | 15.235 | 1173 | C_9_H_20_O | —^c^ | 6.42 ± 0.02^b^ | 7.90 ± 1.29^b^ | 11.43 ± 2.90^a^ |
| Levomenthol | 15.485 | 1182 | C_10_H_20_O | —^c^ | 14.11 ± 2.12^a^ | 13.25 ± 1.72^a^ | 9.56 ±0.00^b^ |
| α-Terpineol | 15.967 | 1198 | C_10_H_18_O | 3.39 ± 0.61^a^ | 3.87 ± 0.00^a^ | —^b^ | —^b^ |
| Geraniol | 17.533 | 1254 | C_10_H_18_O | —^c^ | ^—c^ | 11.93 ± 2.99^b^ | 24.6 ± 6.73^a^ |
| 2,4-Decadien-1-ol | 19.511 | 1327 | C_10_H_18_O | —^c^ | 7.75 ± 1.05^a^ | 5.05 ± 0.25^b^ | 6.32 ± 2.62^ab^ |
| **Ketone** |  | | | | | | |
| Acetoin | 3.313 | <800 | C_4_H_8_O_2_ | —^b^ | 8.72 ± 0.73^a^ | —^b^ | —^b^ |
| 6-Methyl-5-hepten-2-one | 9.677 | 982 | C_8_H_14_O | 10.52 ± 2.32^c^ | 33.17 ± 1.19^a^ | 25.07 ± 0.48^b^ | 25.99 ± 4.42^b^ |
| 6-Methyl-3,5-heptadiene-2-one | 13.223 | 1104 | C_8_H_12_O | 6.95 ± 0.85^c^ | 20.99 ± 2.07^a^ | 15.85 ± 1.79^b^ | 16.89 ± 2.29^b^ |
| 2-n-Hexylcyclopentanone | 19.876 | 1341 | C_11_H_20_O | 2.95 ± 0.46^b^ | 4.23 ± 0.79^b^ | 4.27 ± 0.71^b^ | 6.96 ± 1.85^a^ |
| (E)-1-(2,6,6-Trimethyl-1,3-cyclohexadien-1-yl)-2-buten-1-one | 20.984 | 1383 | C_13_H_18_O | —^c^ | —^c^ | 3.96 ± 1.45^b^ | 5.20 ± 0.07^a^ |
| (E)-6,10-Dimethyl-5,9-undecadien-2-one | 22.668 | 1449 | C_13_H_22_O | 2.90 ± 0.70^b^ | 6.28 ± 2.93^a^ | 4.75 ± 0.60^a^ | 5.09 ± 0.62^a^ |
| trans-β-Ionone | 23.478 | 1482 | C_13_H_20_O | 24.38 ± 3.49^b^ | 34.52 ± 5.78^ab^ | 29.11 ± 2.57^b^ | 38.67 ± 6.00^a^ |
| (R)-5,6,7,7a-Tetrahydro-4,4,7a-trimethyl-2(4H)-benzofuranone | 24.712 | 1533 | C_11_H_16_O_2_ | 12.97 ±1.8 ^ab^ | 13.05 ± 3.84^ab^ | 9.84 ± 1.18^b^ | 16.92 ± 2.73^a^ |
| **Aldehydes** |  | | | | | | |
| Hexanal | 4.813 | <800 | C_6_H_12_O | 6.17 ± 0.36^a^ | 4.53 ± 0.84^b^ | —^c^ | 4.27 ± 0.13^b^ |
| (E)-2-Heptenal | 8.864 | 953 | C_7_H_12_O | —^c^ | 3.76 ± 0.37^a^ | —^c^ | 3.12 ± 0.49^b^ |
| Benzaldehyde | 9.037 | 959 | C_7_H_6_O | 4.63 ± 0.29^b^ | 10.14 ± 1.12^a^ | 5.55 ± 0.96^b^ | 9.36 ± 2.06^a^ |
| (E,E)-2,4-Heptadienal | 10.487 | 1011 | C_6_H_8_O | 3.49 ± 0.11^c^ | 7.68 ± 0.70^a^ | 1.66 ± 0.00^d^ | 4.67 ± 0.60^b^ |
| Benzeneacetaldehyde | 11.471 | 1044 | C_8_H_8_O | 2.29 ± 0.31^d^ | 15.87 ± 1.98^a^ | 7.15 ± 0.89^c^ | 10.49 ± 0.33^b^ |
| (E)-2-Octenal | 11.895 | 1059 | C_8_H_14_O | 7.61 ± 1.11^b^ | 16.31 ± 4.77^a^ | 11.79 ± 1.70^ab^ | 13.86 ± 5.42^a^ |
| Nonanal | 13.293 | 1107 | C_9_H_18_O | 6.77 ± 1.40^a^ | —^b^ | —^b^ | —^b^ |
| (E)-2-Nonenal | 14.913 | 1162 | C_9_H_16_O | 8.37 ± 1.56^c^ | 18.20 ± 1.38^a^ | 10.62 ± 1.31^c^ | 14.05 ± 2.48^b^ |
| 2,6,6-Trimethyl-1,3-cyclohexadiene-1-carboxaldehyde | 16.09 | 1202 | C_10_H_14_O | 11.50 ± 2.28^ab^ | 12.35 ± 0.12^ab^ | 9.15 ± 1.15^b^ | 14.80 ± 2.47^a^ |
| 3,5-Dimethyl-benzaldehyde | 16.561 | 1219 | C_9_H_10_O | 19.68 ± 1.65^c^ | 48.59 ± 1.17^b^ | 52.44 ± 4.16^b^ | 69.79 ± 4.48^a^ |
| 2,6,6-Trimethyl-1-cyclohexene-1-carboxaldehyde | 16.66 | 1223 | C_10_H_16_O | 9.90 ± 4.07^a^ | —^b^ | —^b^ | —^b^ |
| (E,E)-2,4-Decadienal | 19.353 | 1321 | C_10_H_16_O | 11.89 ± 1.98^b^ | 16.29 ± 1.85^a^ | 11.79 ± 1.43^b^ | 14.87 ± 2.14^ab^ |
| trans-4,5-Epoxy-(E)-2-decenal | 20.975 | 1382 | C_10_H_16_O_2_ | —^b^ | 3.94 ± 0.35^a^ | —^b^ | —^b^ |
| **Esters** |  | | | | | | |
| Methoxy-phenyl-oxime | 7.241 | 892 | C_8_H_9_NO_2_ | —^b^ | —^b^ | —^b^ | 14.16 ± 4.02^a^ |
| Formic acid heptyl ester | 9.241 | 966 | C_8_H_16_O_2_ | —^b^ | —^b^ | —^b^ | 3.81 ± 0.00^a^ |
| Dihydro-5-pentyl-2(3H)-furanone | 20.439 | 1362 | C_9_H_16_O_2_ | —^b^ | —^b^ | —^b^ | 6.48 ± 0.00^a^ |
| 1,2-Benzenedicarboxylic acid bis(2-methylpropyl) ester | 31.765 | 1862 | C_16_H_20_O_4_ | 3.75 ± 0.46^a^ | 5.68 ± 1.83^a^ | 4.33 ± 0.46^a^ | 4.97 ± 2.02^a^ |
| Hexadecanoic acid methyl ester | 32.775 | 1928 | C_17_H_34_O_2_ | —^c^ | 3.25 ± 0.18^b^ | 3.20 ± 0.24^b^ | 5.49 ± 0.16^a^ |
| Hexadecanoic acid ethyl ester | 33.691 | 1995 | C_18_H_36_O_2_ | 4.08 ± 1.15^b^ | 4.78 ± 0.65^b^ | 4.71 ± 0.86^b^ | 8.31 ± 0.85^a^ |
| **Terpenes** |  | | | | | | |
| 2,3-Nonadiene | 10.729 | 1019 | C_8_H_14_ | —^b^ | 6.49 ± 1.07^a^ | —^b^ | —^b^ |
| 3-Ethyl-2-methyl-1,3-hexadiene | 11.09 | 1032 | C_9_H_16_ | 15.94 ± 0.3^b^ | 19.88 ± 0.90^a^ | 10.26 ± 1.32^c^ | 11.46 ± 1.51^c^ |
| (E,E)-2,6-Nonadienal | 14.697 | 1155 | C_9_H_14_O | 1.51 ± 1.31^b^ | 9.29 ± 0.87^a^ | —^c^ | —^c^ |
| **Phenols** |  | | | | | | |
| 2-Methoxy-phenol | 12.693 | 1086 | C_7_H_8_O_2_ | —^c^ | 10.65 ± 1.62^a^ | 6.44 ± 1.26^b^ | 9.94 ± 1.40^a^ |
| 2,5-Dimethyl-phenol | 14.562 | 1150 | C_8_H_10_O | —^c^ | 14.28 ± 2.02^a^ | 9.39 ± 0.68^b^ | 12.3 ± 2.40^ab^ |
| 2,4,5-Trimethyl-phenol | 17.938 | 1269 | C_9_H_12_O | —^c^ | 8.29 ± 0.43^b^ | 9.09 ± 0.25^b^ | 12.53 ± 0.69^a^ |
| 2-Methoxy-4-vinylphenol | 19.132 | 1312 | C_9_H_10_O_2_ | 9.83 ± 2.21^c^ | 61.83 ± 1.66^a^ | 53.25 ± 2.54^b^ | 67.86 ± 5.22^a^ |
| 2,4-Di-tert-butylphenol | 24.109 | 1507 | C_14_H_22_O | 6.34 ± 1.04^a^ | —^b^ | —^b^ | 5.64 ± 0.00^a^ |
| **Others** |  | | | | | | |
| 2-Pentyl-furan | 9.865 | 989 | C_9_H_14_O | 3.63 ± 1.19^c^ | 31.66 ± 6.36^b^ | 23.38 ± 4.66^b^ | 42.25 ± 4.55^a^ |
| Tetramethyl-pyrazine | 12.658 | 1085 | C_8_H_12_N_2_ | 9.83 ± 3.04^a^ | —^b^ | —^b^ | —^b^ |
| Naphthalene | 15.688 | 1189 | C_10_H_8_ | —^b^ | —^b^ | —^b^ | 28.5 ± 2.43^a^ |
| 4-(9-Borabicyclo[3.3.1]non-9-yloxy)-2-thiapentane | 16.363 | 1212 | C_12_H_23_BOS | 3.76 ± 0.91^c^ | 5.69 ± 0.27^bc^ | 7.26 ± 0.69^b^ | 13.61 ± 2.14^a^ |
| 2,3-Dihydro-2,2-dimethyl-7-benzofuranol | 18.718 | 1297 | C_10_H_12_O_2_ | 4.53 ± 0.93^c^ | 13.07 ± 2.20^b^ | 12.74 ± 0.57^b^ | 17.63 ± 2.14^a^ |
| N,N-Dibutyl-formamide | 18.84 | 1301 | C_9_H_19_NO | 4.10 ± 0.53^d^ | 8.12 ± 0.87^c^ | 10.95 ± 0.89^b^ | 17.72 ± 1.65^a^ |
| 4-(1H-Pyrazol-1-yl)benzeneamine | 21.145 | 1389 | C_9_H_9_N_3_ | —^b^ | —^b^ | —^b^ | 6.49 ± 0.03^a^ |

The data were expressed as the mean value ± standard deviation. The data with different superscript letters (a-d) in the same row indicate significant difference (P ≤ 0.05), and '—' means not detected.
